# Sustainable AGP alternatives: a systems approach to non-antibiotic growth regulators standardization, synergistic formulation and environmental safety

**DOI:** 10.3389/fvets.2025.1695160

**Published:** 2026-01-30

**Authors:** Manzar Abbas, Ghulam Abbas, Fatima, Abdullah Hassan Hashmi, Seemab Jaffery, Yunxia Li, Gaoping Zhao, Li Xihe

**Affiliations:** 1 Inner Mongolia Saikexing Institute of Breeding and Reproductive Biotechnology in Domestic Animal, Hohhot, China; 2 National Center of Technology Innovation for Dairy, Hohhot, China; 3 Department of Animal Production, Riphah College of Veterinary Sciences, Riphah International University, Lahore, Pakistan; 4 Key Laboratory of Animal Genetics, Breeding and Reproduction of Shaanxi Province, College of Animal Science and Technology, Northwest A&F University, Yangling, China; 5 University Institute of Food Science and Technology, University of Lahore, Lahore, Pakistan; 6 Faculty of Agriculture, University of Agriculture, Faisalabad, Pakistan; 7 Research Center for Mongolian Genetic Resources of Plateau Animal and Teaching Center for Experimental Biology, School of Life Sciences, Inner Mongolia University, Hohhot, Inner Mongolia, China; 8 State Key Laboratory of Reproductive Regulation and Breeding of Grassland Livestock, Inner Mongolia University, Hohhot, Inner Mongolia, China

**Keywords:** antibiotic alternatives, phytogenic feed additives, antimicrobial peptides, IgY antibody applications, bacteriophage therapy, symbiotic

## Abstract

Growing consumer preference for livestock products labeled “*Raised without Antibiotics*” *(RWA)* or “*No Antibiotics Ever*” *(NAE)*, escalating crisis of antimicrobial resistance due to long use of antibiotic growth promoters (AGPs) along with stringent regulatory restrictions, has intensified the demand for sustainable alternatives. This review summarizes recent advances in non-antibiotic strategies to enhance livestock production while aligning with global regulatory bans on in-feed antibiotics. We first delineate the multifunctional mechanisms of AGPs, primarily through gut microbiota modulation and immunomodulation, to establish a benchmark for alternative efficacy. The core analysis critically evaluates leading antibiotic substitutes, including probiotics, prebiotics, synbiotics, organic acids, dietary enzymes, and phytogenic food additives (PFAs). Among all, PFAs rich in terpenoids and phenolics for their antimicrobial, antioxidant, and gut health promoting properties along with cost-efficiency, scalability, and one health implications are preferred alternative to antibiotics. Further, we underscore emerging technologies such as antimicrobial peptides (AMPs), hyper-immune egg yolk antibodies (IgY), bacteriophages, genomic medicines, and clays and trace minerals, highlighting commercially approved examples like bacteriophage to control *Salmonella*. Despite demonstrated success in improving feed efficiency, growth performance, and overall animal health, challenges regarding consistency, bioavailability, and regulatory approval persist. The conclusive evidence positions a strategic combination of these natural and advanced alternatives, particularly optimized PFA formulations, as a viable and sustainable pathway to achieving antibiotic-free animal husbandry, thereby mitigating AMR risks and ensuring future food security.

## Graphical abstract

**Figure:**
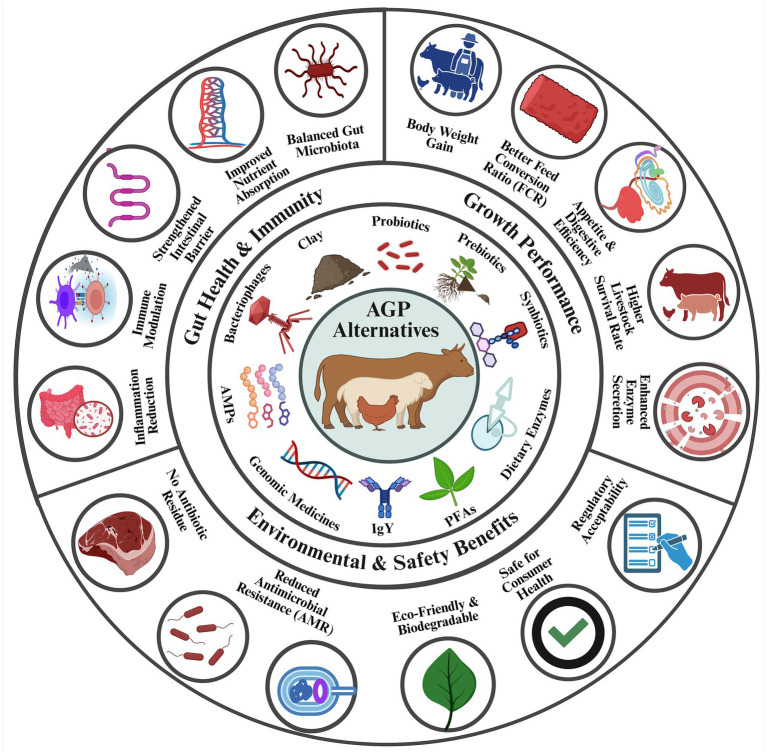

## Introduction

Antibiotics are extensively supplemented in animal feed to enhance livestock productivity by improving feed efficiency, preventing and controlling infections, and reducing mortality (1). However, their use poses significant drawbacks, including the potential to drive antimicrobial resistance and jeopardize human health (259). AMR represents a critical public health challenge due to the emergence, transmission, and persistence of multidrug resistance (MDR) pathogens across animal, human, and environmental ecosystem. AMR is responsible for ~ 700,000 human deaths annually, with projections suggesting their figure could escalate to 10 million per year by 2050 (2). Recognizing the gravity of this threat, the WHO issued guidelines in 1997 and EU prohibited use of antibiotics as growth promoters in 2006 (3). In US, increasing consumer awareness and demand for antibiotic-free animal products have intensified scrutiny of antibiotic use in livestock (4). In response, the US Food and Drug Administration (FDA) mandated in 2013 (Guidance for Industry #213) that major animal pharmaceutical companies cease labeling antibiotics for growth promotion and instead require veterinary oversight for therapeutic applications. This regulatory shift was further reinforced by the implementation of the Veterinary Feed Directive (VFD) in 2015 (5). Over the past two decades, research into alternative antimicrobials – particularly plant-derived antibiotic substitutes – has expanded significantly as a strategy to reduce reliance on antibiotics in animal husbandry. The WHO identifies >1,340 plants with antimicrobial properties, yielding >30,000 bioactive compounds (6, 7). These secondary metabolites—terpenoids, phenolics, and alkaloids—serve ecological roles (e.g., pest resistance) and offer potential as AGP alternatives. Extensive evaluation of AGPs prior to administration must extend beyond zootechnical parameters such as weight gain, and FCR to include their potential for AMR mitigation. This review proposes integration of natural and advanced antibiotic alternatives as potential growth promoters to constitute a viable and sustainable antibiotic-free animal husbandry.

## Mechanism of action of antibiotic growth promoters

**Figure 1:**
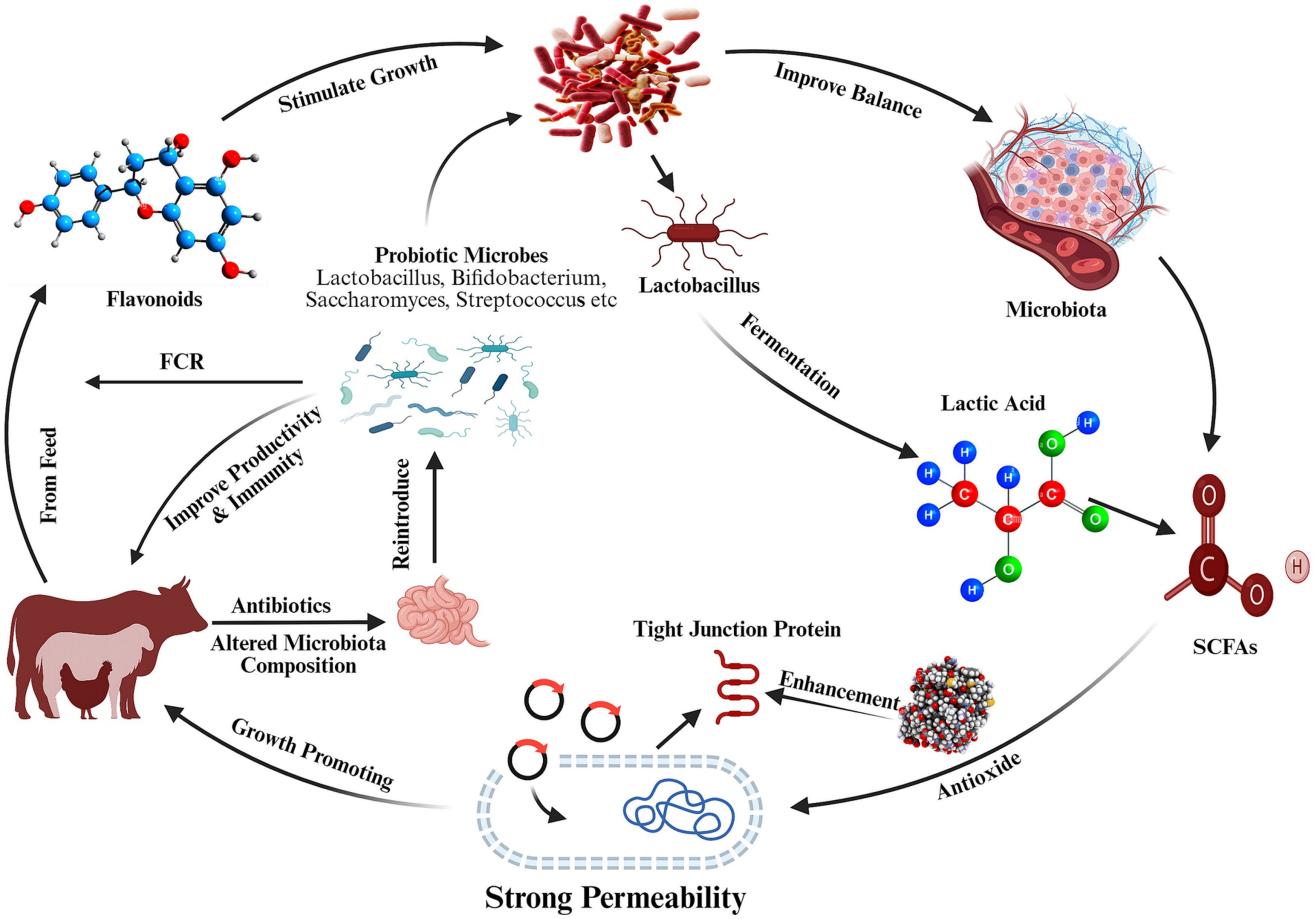
Multifunctional role of Lactobacillus and other probiotics in gut homeostasis. Probiotics antagonize pathogens via the secretion of antibacterial agents and organic acids, modulate the host’s microbiota, fostering a symbiotic community that produces health-promoting metabolites like SCFAs. Strengthen gut epithelial barrier for intestinal permeability and reduced inflammation.

Understanding the mechanism of action of AGPs is essential for identifying effective antibiotic alternatives. AGPs reduce gut microbiota diversity and abundance, diminishing competition for nutrients and suppressing microbial metabolites that impair growth, such as those involved in bile and amino acid catabolism (8). However, Knarreborg et al. (9) challenged his hypothesis, proposing that AGPs primarily interact with host-immune cells rather than directly inhibiting microbiota. AGPs may mitigate inflammation and cytokines release, which otherwise suppress appetite and increase muscle catabolism (Figure 1), thereby improving energy utilization and growth efficiency (10). Advances in molecular biology and bioinformatics indicate that AGPs modulate gut-microbiota (11), reduce inflammation, enhance nutrient absorption, and optimize growth performance (12). Specifically, Lin (13) demonstrated that AGPs decrease bile salt hydrolase (BSH) – producing bacterial, altering bile acid metabolism and host lipid utilization.

Mouse studies reveal that sub-therapeutic antibiotics reshape gut microbiota composition, favoring microbes with enhanced carbohydrate-to-SCFA (short chain fatty acids) metabolic pathways (14). Notably, this growth-promoting phenotype was transmissible to germ-free hosts, confirming microbiota-mediated effects rather than direct antibiotic action. Early-life low-dose antibiotic exposure also induces lasting metabolic changes by accelerating age-related microbiota shifts and altering ileal immune gene expression (15). While findings in rodents may not directly translate to livestock, they provide insights into potential AGP mechanisms.

## Classes of antibiotic alternatives

An ideal AGPs should enhance nutrient absorption, maximize animal performance, and replicate AGP benefits (12). Effective substitutes must also modulate immunity while improving feed efficiency and growth, aligning with proposed AGP mechanisms (16). In poultry production, tested alternatives include probiotics, prebiotics, phytobiotics, bacteriophages, synbiotics, organic acids, enzymes, phytogenics, and metals (17). Emerging options – such as hyperimmune egg yolk IgY (18), antibacterial peptides (AMP) (19), bacteriophages (20), and clay minerals (21) – have also gained attention for their potential as AGP replacements.

### Probiotics

**Figure 2:**
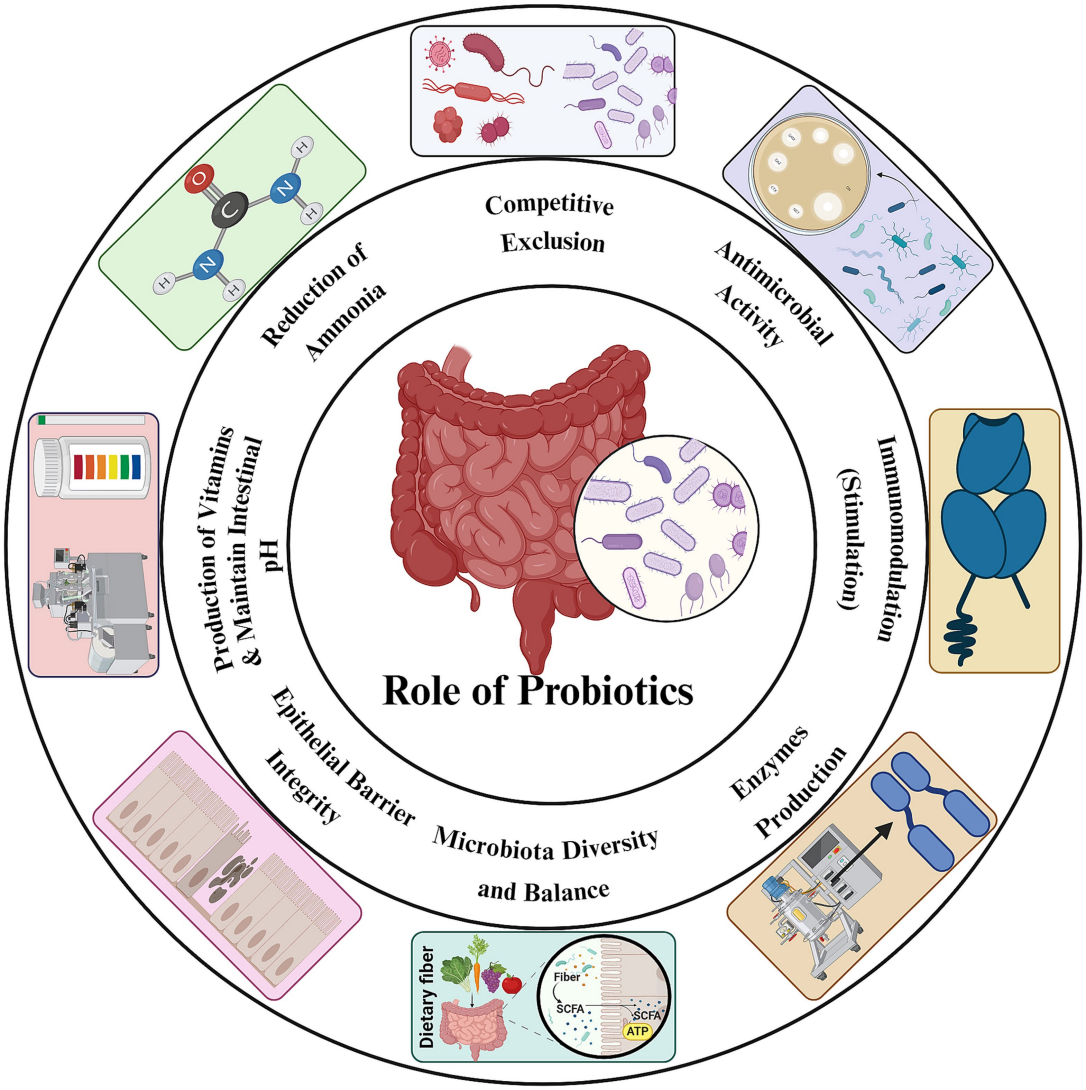
Dual-pathway model of probiotic function. (1) Competitive exclusion – involving pathogen inhibition via antimicrobial activity, ammonia reduction, and pH control; and (2) Host interaction, enhances barrier function, provides enzymatic and nutritional support, and stimulates immune responses.

Probiotics or direct-fed microbial (DFMs) represent one of the most promising alternatives to AGPs (22). When administered in feed – either alone or in combination with other additives – probiotics confer health benefits to the host (Figure 2). Probiotics are administered *in-ovo* or spraying on 1 day old chicks (23). Livestock probiotics primarily utilize *Bacillus*, *Bifidobacterium*, *Enterococcus*, *Lactobacillus*, *Streptococcus*, *Lactococcus*, and *Saccharomyces* spp. (24, 25). *Lactobacillus* strains (single or multi-strain) improve animal body weight and feed conversion ratio (FCR) (26). *Bacillus coagulans*, *Bacillus subtilis*, *Bacillus licheniformis*, and *Bacillus amyloliquefaciens* boost productivity (27). Other effective probiotic strains are *Rhodopseudomonas palustris* (28), *Enterococcus faecium* (29), and *Clostridium butyricum* (30). Spray application by Faria Filho et al. (31) reported a 0.14 increase in body weight and 0.10-point FCR reduction across 27 Brazilian trials. Similarly, spray application by Blajman et al. (32) confirmed improved FCR and weight gain, noting superior efficacy when administered via water versus feed.

Probiotics enhance performance through microbial modulation, gut health, immune enhancement, and nutrient utilization (33). In microbial modulation, competitive exclusion of pathogens, production of antimicrobial compounds against pathogens (e.g., bacteriocins, SCFAs), and pH reduction (34). Probiotics improve gut health which plays pivotal role in improvement of villus height, crypt depth (35), and enrichment of beneficial microbiota such as *Lactobacillus* and *Bifidobacterium* (36). Probiotics improve livestock immunity by elevating secretory IgA, macrophage activation, and cytokine production (37) as well as enhance digestive enzyme activity and reduce anti-nutritional compounds (38). Efficacy of probiotics varies by strain, dosage, and environmental factors (39). Optimal probiotic strains must resist gastric acidity, adhere to intestinal epithelium, and exhibit immunomodulatory properties (40). To ensure viability and colonization in the intestine encapsulation techniques such as micro-encapsulation can improve viability of probiotics (41). Despite benefits, the propensity of probiotic *Enterococcus* and *Lactobacillus* strains for horizontal gene transfer (HGT), mediated by their mobile genetic elements (MGEs), poses a significant risk of amplifying the gut resistome demanding rigorous safety screening before commercialization of probiotic strains (42).

### Prebiotics

**Figure 3:**
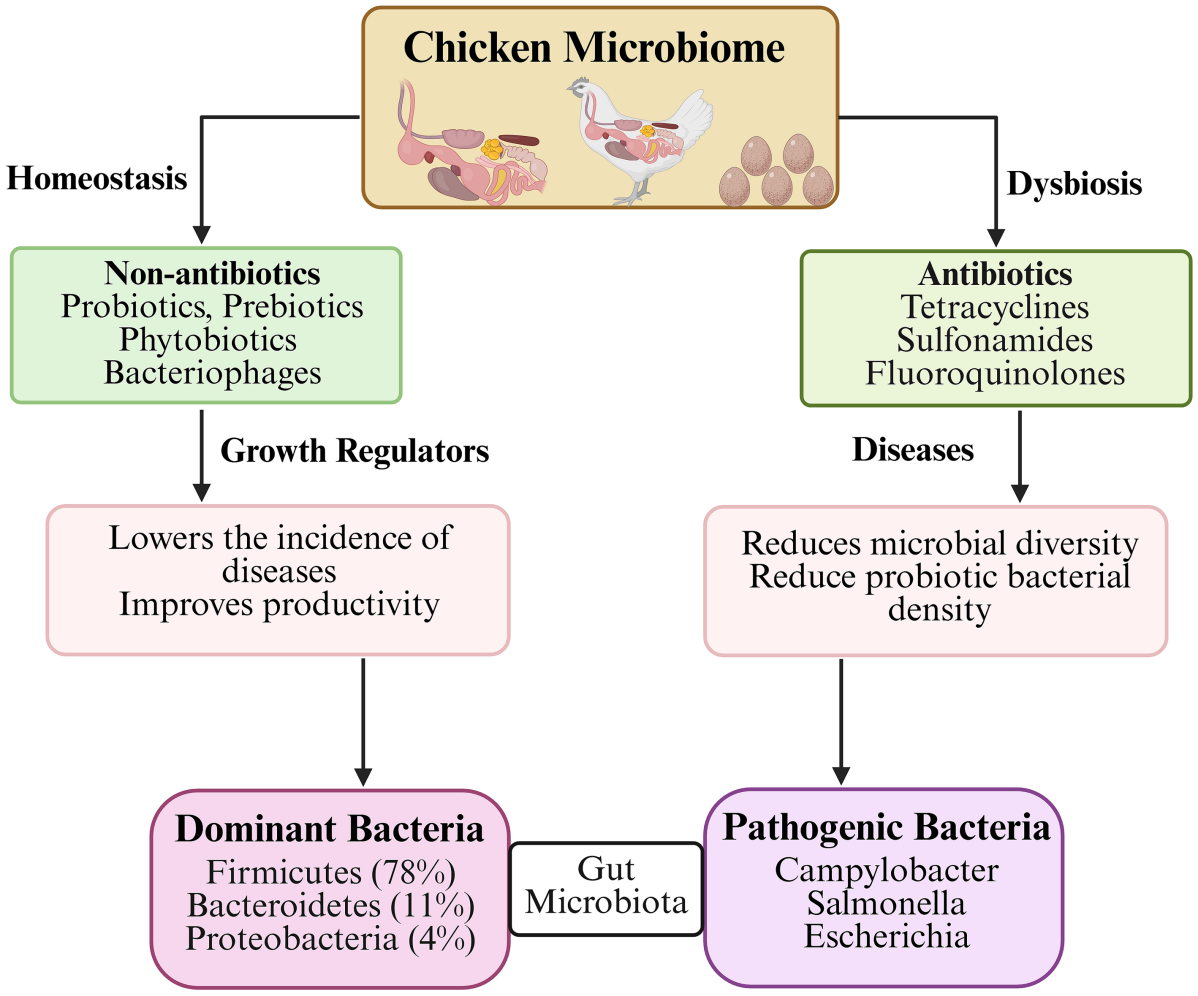
Schematic representation of factors modulating gut microbiome and consequent health out-comes. Chicken microbiome is dominated by Firmicutes (78%), Bacteroidetes (11%), and Proteobacteria (4%). Probiotics, prebiotics, phytobiotics, and bacteriophages maintain homeostasis by outcompeting pathogens resulting in improved disease resistance and productivity. While broad-spectrum antibiotics depletes beneficial microbes.

**Figure 4:**
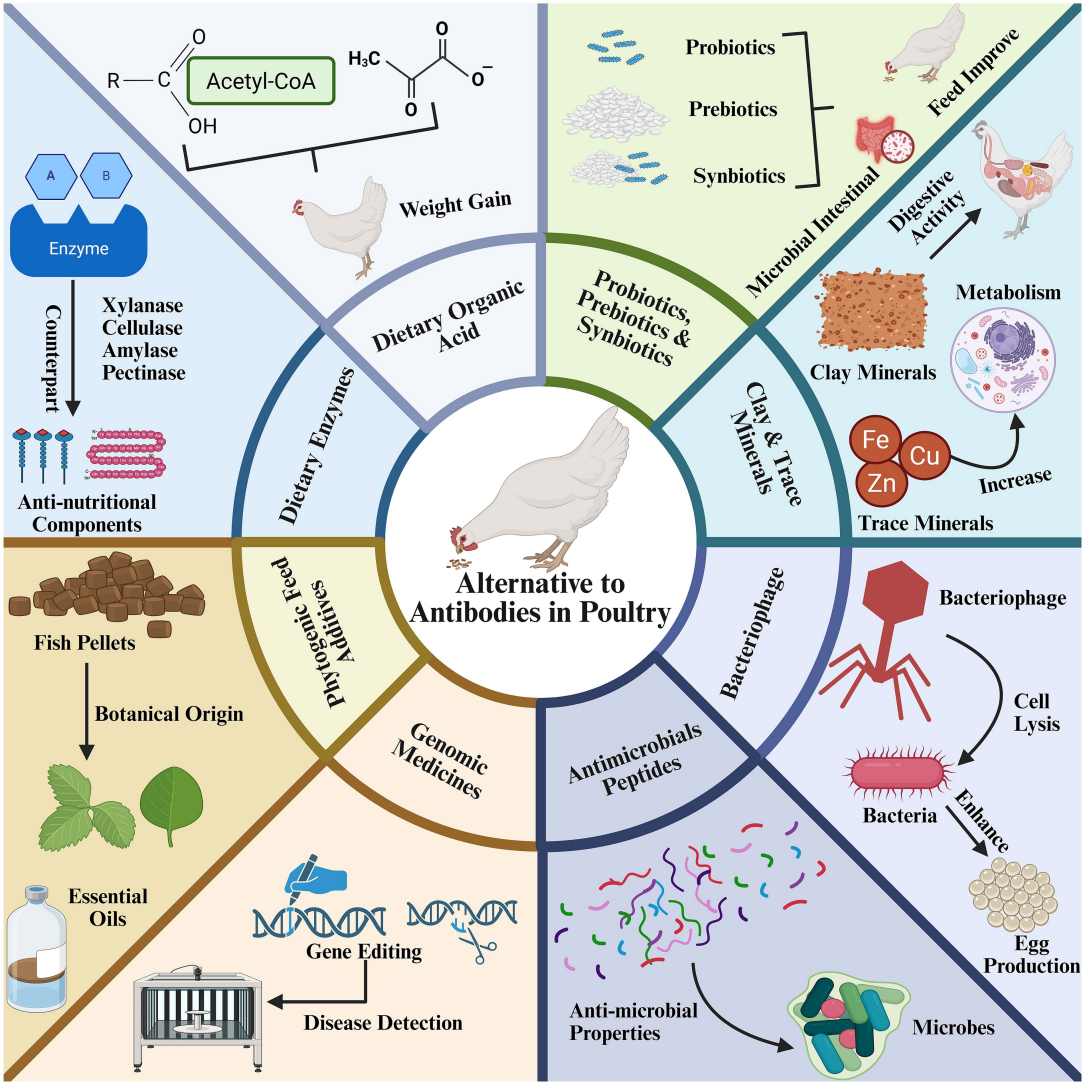
Comprehensive schematic overview of antibiotic alternatives in poultry production. Enzymes (e.g., xylanase, cellulase) counteract anti-nutritional factors; dietary organic acids, probiotics, prebiotics, and synbiotics foster beneficial microbiota; clay and trace minerals support microbial and metabolic functions; bacteriophages kill pathogens to balance microbiota; antimicrobial peptides disrupt membranes of pathogens; genomic medicines enable disease resistance through gene editing; phytogenic additives boost natural growth and immune support; and essential oils delivering antimicrobial and antioxidant effects, collectively improve feed efficiency and gut health resulted in more weight gain.

Prebiotics or fermentable oligosaccharides constitute a well-established alternative to AGPs (43). When incorporated into feed—either as standalone supplements or synergistically with probiotics, prebiotics selectively modulate intestinal microbial composition by selectively stimulating commensal bacterial growth enhancing host health and performance (Figure 3). Prebiotics are non-digestible feed components that confer health benefits by selectively stimulating the growth or activity of beneficial intestinal bacteria (44, 45). These compounds, primarily non-starch polysaccharides (NSP) or oligosaccharides (*β*-glucans)—including mannan-oligosaccharides (MOS) (46), fructo-oligosaccharides (FOS) (47), galactooligosaccharides (GOS) (48), lactulose (49), arabinoxylans (50), xylooligosaccharides (XOS) (51), isomalto-oligosaccharides (IOS) (52), and inulin (53) – are derived from microbial or plant sources which selectively enhance beneficial gut microbiota (Figure 4). Yeast cell-wall supplements relatively raised body weight by 1.61% and reduced FCR by 1.99% (54). Prebiotics administration relatively improved body weight (5.41%), FCR (2.54%), and mortality rates (10.5%) (55).

Prebiotics enhance livestock performance through microbial modulation, metabolic effects, and immunomodulation (56). MOS is derived from *Saccharomyces cerevisiae* cell walls, acts as a pathogen-associated molecular pattern (PAMP), triggering innate immune responses (57), enhances body weight gain, FCR, intestinal villus height (58, 59), and immune competence (60). FOS improves broiler efficiency (61), while IOS enhance weight gain and FCR (26). Lactulose supplementation increases body weight, FCR, villus height, goblet cell density, SCFA production, and *Lactobacillus* populations (62, 63). Fermentation yields SCFAs (e.g., acetate, butyrate), which fuel enterocytes and maintain gut barrier integrity (57). Ideal prebiotics must resist gastric acidity, evade enzymatic hydrolysis, and avoid mucosal absorption (64).

### Synbiotics

Synbiotics, defined as nutritional supplements combining probiotics and prebiotics, function synergistically to enhance host health by improving probiotic survival, implantation, and the selective stimulation of beneficial intestinal bacteria (65). Dietary supplementation with synbiotics has been shown to increase body weight, average growth rate, feed efficiency, and carcass yield compared to control or probiotic-only diets (66). Further corroborating these findings, Boostani et al. (67) reported similar growth improvements, while Mohnl et al. (68) observed a 2.04% increase in body weight and a 0.9% reduction in mortality. Mookiah et al. (26) noted enhanced weight gain and reduced FCR in birds fed a symbiotic blend of 11 *Lactobacillus* strains, and isomalto-oligosaccharides (IOS), though no twofold synergistic effect was observed compared to individual components. Yitbarek et al. (69) documented greater weight gain in pullets supplemented with probiotics and yeast-derived carbohydrates versus prebiotic-only or control group. However, some studies found no significant performance improvement with synbiotic inclusion (70). Beyond growth metrics, synbiotic have been shown to enhance gut morphology, increasing villus height and crypt depth (71). Their potential as antibiotic alternatives in poultry production lies in optimizing performance and reducing intestinal pathogen load. Nevertheless, careful selection of compatible prebiotic-probiotic combinations and rigorous validation of synergistic efficacy – relative to standalone use – are essential for maximizing their benefits.

### Dietary organic acids (DOAs)

Dietary organic acids (DOAs) are historically recognized as antimicrobial agents and considered viable alternatives for AGPs in livestock production. Chemically, these acids are classified as either mono-carboxylic acids (e.g., formic, acetic, propionic, butyric) or hydroxyl-group-containing carboxyl groups (Table 1) (72). Naturally occurring in plant and animal tissues, some – particularly short-chain fatty acids (SCFA) – are also produced in the hindgut through microbial fermentation of carbohydrates (73). DOAs can be administered via feed or water, either as free acids, salts, or blended formulations (12). Supplementation with fumaric acid improved feed efficiency and weight gain in broilers (74), while butyric acid enhanced growth rates (75). Other DOAs are citric (76), formic (77), malic (78), sorbic (79), and tartaric acids (80), have also demonstrated efficacy in livestock. Blends (OABs) leverage synergy, broader spectrum of activity, and yield superior results as compared to single DOA. For example, chromium OAB improved broiler meat quality by decreasing fat content per 100 g of carcass, increased total ash and protein accretion, high plasma chromium concentration, reduced pH in the gizzard and duodenum, and deposition of chromium in breast and thigh as compared to single DOA administration (81).

**Table 1:** Organic acids as alternative to antibiotics.

Organic acid	Species	Pathogen/Physiology	Findings	References
Formic acid	White Leghorn	Effect of in 68-week-old	1 or 1.5 mL/L: ↑ egg number, weight and grading, quality of shell and immunity	(161)
Formic acid and blends with sodium formate and propionic acid	Poultry	Four *Salmonella* strains	Blend of formic acid and propionic acid ↓ pathogens	(162)
Citric, propionic, or acetic acid	Broiler	*Listeria monocytogenes*	2%: propionic or acetic acid ↓ growth	(163)
Fumaric acid, lactic acid, and butyric acid	Broiler	Performance, blood chemistry and intestinal histomorphology	2 and 3%: ↑ small intestinal villus height serum Ca and P levels	(164)
Citric acid	Male broilers	Growth, Digestion, Linear body	3%: ↑ nutrient digestibility in the ileum, performance, and retention of minerals	(165)
Sodium butyrate	Broiler	Growth, Performance, Immunity and *E. coli* LPS	1 g/kg: ↑ growth, moderate immunity, ↓ tissue damage	(166)
Aciflex® (Lactic acid, citric acid, CuSO_4_, phosphoric acid)	Broiler	*Salmonella* and *E. coli*	2%: ↑ body weight, FCR, carcass yield and liver weight; ↓ pathogens	(167)
Formic acid mixed with sodium formate	Broiler	Growth, nalidixic acid–resistant and *Salmonella enterica* (Typhimurium)	0.9%: Dose-dependent ↑ body-weight, ↓ pathogens	(168)
Formic acid	Broiler	*Campylobacter coli*	0.08%: ↓ pathogens in vivo	(169)
Propionic, formic acids and their salts	Broiler	Growth, intestinal morphology, cecal microbes, Immunity and *E. coli* (K88)	↑ growth, morphology, cecal microbes, and immunity	(170)
Short- and medium-chain fatty acid and phenolic compounds	Male parental chicks	Intestinal integrity and pH, caecal microbiota, caecal SCFA	All blends offered similar intestinal protection against necrotic enteritis	(171)
Fumaric acid and mixture of Calcium lactate, calcium format, capric and caprylic acids	Weaned Piglets	Growth, gastrointestinal parameters	Acidifier blend in absence of fumaric acid ↑ intestinal weight, jejunum villi height, total coliform and *E. coli* count in cecum	(172)
Caprylic and/or capric acid	Piglets	*C. perfringens*	1–2 g/kg of feed: ↑ growth rate, villus height, protein and fiber digestibility; ↓ pathogens and mortality	(173)
Fumaric acid, lactic acid, caprylic and capric acid, medium chain fatty acids	Weaning Piglets	Changes in gut microbiome	↓ gut pH and *E. coli* virulence genes	(174)
Corn-wheat bran-soybean basal diet and 2 formulations of mixed organic acid	Barrows	Nutrient digestibility, composition of the VFAs, and intestinal microbiome	↑ digestion, volatile FA concentration, and intestinal flora	(175)
Acetic, phosphoric, lactic, fumaric, tartaric acid	Quail	*Salmonella enteritidis*	3 mL/L: ↓ *in vivo* pathogens	(176)
Disodium fumarate	Goat	CH_4_ emission, fermentation and bacterial counts	10 g/day: ↑ in vivo fermentation, 11.9% ↓ CH_4_ emission, ↓ pathogens	(177)
Malic and fumaric acid with plant leaves	Ruminants	to improve their usefulness as substitute feeds	0.1% fumaric acid with *Camellia sinensis*: ↑ digestibility, ↓ protozoan	(178)
Lauric acid or coconut oil	Holstein cows	Protozoa, fermentation, digestion, omasal nutrient flow, and milk quantity	40% ↓ of Protozoa; not sufficient to improve nutrient utilization	(179)
Lauric acid and myristic acids	Lactating dairy cows	Fermentation, FA profile of milk, and microbial and protozoal counts	240 g/cow/day: Lauric acid ↑ ruminal fermentation, ↓ pathogens	(180)
Organic acid and botanical blend (OABP)	Ruminants	*S. enterica* (Typhimurium) and *E. coli*	↓ pathogens count by ≥5%	(181)

The antimicrobial mechanisms of DOAs, though not fully elucidated, may involve lowering gastrointestinal pH, altering stomach mucosa physiology (77), modulating gut microbiota by directly suppressing pathogens (via cell-wall penetration) or indirectly favoring acid-tolerant beneficial bacteria (*Lactobacillus*) while reducing nutrient competition (82), enhancing nutrient digestibility through improved enzyme activity (83), promoting gut health via direct epithelial stimulation (e.g., SCFAs as an energy source for enterocyte proliferation). Despite their benefits, inconsistent efficacy, palatability and stability attributed to factors such as inclusion rates, organic acid sources, and dietary buffering capacity (84). For example, formic, propionic, acetic and butyric acids have pungent smell and low palatability and stability while lactic acid has better palatability and stability (85). Formulation of OABs by addition of salts such as calcium formate and potassium sorbate mask pungent smell and deliver the benefits of DOAs without palatability drawbacks (86). Further research is needed to optimize their use and establish reliable mechanisms of action, ensuring their effectiveness as sustainable antibiotic alternatives.

### Dietary enzymes (DEs)

Dietary enzymes are biologically active proteins that catalyze the breakdown of complex nutrients into absorbable components, enhancing digestive efficiency and nutrient utilization (87). Primarily derived from microbial fermentation, these exogenous enzymes – including phytases, carbohydrates (xylanase, cellulase, *α*-galactosidase, *β*-mannanase, α-amylase, pectinase), and proteases – are widely employed in swine diets. Their primary role involves counteracting anti-nutritional factors (ANFs) inherent in plant-based feed ingredients, such as phytic acid, non-starch polysaccharides (NSP), and cell-wall complexes, thereby improving nutrient bioavailability (88). Meta-analysis revealed mixture of phytase and NSP-degrading enzymes increased body weight by 3.73% and reduced FCR by 2.64% (89). Similarly, β-mannanase supplementation improved weight by 4.8% and FCR by 4.2 points in market-age broilers (90). Combined xylanase, amylase, and protease enhanced crude protein, starch, and fat digestibility by 22.7, 88.9, and 33.4%, respectively (91). Dietary enzymes represent a promising alternative to antibiotics, enhancing nutrient utilization and gut health. However, optimizing their application requires tailored formulations to account for dietary and biological variability (40). Further research should focus on standardized protocols to maximize consistency in poultry production systems.

The efficacy of in-feed enzymes is attributed to disruption of cell-wall matrices, hydrolysis of indigestible substages, inactivation of ANFs, solubilization of insoluble NSPs, and modulation of gut microbiota (92). Dietary enzymes break cell wall to liberate encapsulated starch, animo acids, and minerals and hydrolyze phytate-P by phytase (92). Dietary enzymes reduce interference of ANFs with nutrient absorption, enhance cecal fermentation of NSPs and SCFA production as well as alter microbial ecology via generation of prebiotic OS and reduction of undigested substrates (40).

### Phytogenic feed additives (PFAs)

Phytogenic feed additives (PFAs) are comprised of bioactive plant-derived compounds incorporated into livestock diets to enhance productivity (93). PFAs are antimicrobial, antioxidant, immunomodulation, digestive enhancement and play key role in gut morphology optimization (94). PFAs directly inhibit pathogens and gut microbiota modulation, reducing microbial toxins and intestinal immune stress (95). Their antioxidative property mitigates oxidative stress, and improve tissue health (96). PFAs augment cytokine production, antibody titers, and immune cell proliferation (97). PFAs improve nutrients digestion via stimulation of pancreatic enzyme secretion, and bile flow (98, 99). Similarly, PFAs increase villus height and transepithelial resistance, improving nutrient absorption and barrier integrity (100, 101). PFAs are derived from thyme, oregano, rosemary, garlic, ginger, cinnamon, and green tea (Table 2). Based on extraction methodology, PFAs are classified as essential oils (e.g., volatile lipophilic compounds obtained via steam/alcohol distillation or cold extraction) and oleoresins (non-aqueous solvent-derived extracts rich in bioactive constituents) (Figure 4) (102). The efficacy of PFAs hinges on their polyphenolic composition, which verities with plant species, anatomical part, geographic origin, harvest timing, storage conditions, and processing techniques (103).

**Table 2:** Active compounds of various herbal plants and their antimicrobial activity.

Common name	Scientific name	Compound	Classic	Activity	Reference
Ashwagandha	*Withania somniferum*	Withaferin A	Lactones	Bacteria, Fungi	(182)
Black Pepper	*Piper nigrum*	Piperine	Alkaloid	Fungi, *E. coli Lactobacillus*	(183)
Ceylon cinnamon	*Cinnamomum verum*	Cinnamaldehyde, Coumarin	Terpenoids, Tannins	General	(184)
Chili peppers	*Capsicum annuum*	Capsaicin	Terpenoid	Bacteria	(185)
Cloves	*Syzygium aromaticum*	Eugenol	Terpenoid	General	(186)
Eucalyptus	*Eucalyptus globulous*	Eucalyptol	Polyphenol, Terpenoids	Bacteria, Viruses	(187)
Fava bean	*Vicia faba*	Thionine	Thionin,	Bacteria	(188)
Garlic	*Allium sativum*	Allicin, Ajoene	Sulfoxides, Sulfated terpenoids	General	(189)
Ginseng	*Panax notoginseng*	Ginsenosides	Saponin	*E. coli, Sporothrix schenckii*, *Staphylococcus*	(190)
Green tea	*Camellia sinensis*	Catechins	Flavonoids	General, *Shigella, Vibrio, S. mutans, Viruses*	(191)
Harmel/Syrian Rue	*Peganum harmala*	β-carbolines	—	Bacteria, Fungi	(192)
Hemp	*Cannabis sativa*	Olivetolic acid	Organic acid	Bacteria and viruses	(193)
Lemon verbena	*Aloysia triphylla*	Volatile compounds	Terpenoid	*E. coli, M. tuberculosis, S. aureus, Ascaris*	(194)
Marigold	*Calendula officinalis*	Carotenoids	Xanthophyll	Bacteria	(195)
Oak	*Quercus rubra*	Tannins, Quercetin	Polyphenols, Flavonoids	General	(196)
Olive	*Olea europaea*	Hexanal	Aldehydes	General	(197)
Onion	*Allium cepa*	Isoalliin	Sulfoxides	Bacteria, *Candida*	(198)
Orange peel	*Citrus sinensis*	Terpene	Terpenoid	Fungous	(199)
Papaya	*Carica papaya*	Latex	Terpenoids, organic acids, alkaloids	General	(200)
Peppermint	*Mentha × piperita*	Menthol	Terpenoid	General	(201)
Potato	*Solanum tuberosum*	Phenolic acids	Flavonoids	Bacteria, Fungi	(202)
Rosemary	*Rosmarinus officinalis*	Camphoriferum	Terpenoid	General	(203)
Thyme	*Thymus vulgaris*	Caffeic acid, Thymol Tannins	Terpenoid, Phenolic alcohol, Polyphenols, Flavones	Viruses, bacteria, fungi	(204)
Turmeric	*Curcuma longa*	Curcumin	Terpenoids	Bacteria, protozoa	(205)

PFAs have gained traction in livestock production as natural alternatives to antibiotic growth promoters (AGPs). For example, a 14-herb mixture significantly improved broiler weight gain and feed efficiency (104). Black cumin (*Nigella sativa*), *Scrophularia striata*, and *Ferulago angulata* demonstrated growth-promoting effects (Table 2) (105). Sugar cane, aniseed, chestnut wood, and *Portulaca oleracea* extracts enhanced body weight gain and reduced FCR (106). Conversely, certain PFAs—such as grape pomace, cranberry extract, and *Macleaya cordata*—showed no measurable impact on performance metrics (107). EOs contain thymol, carvacrol, cinnamaldehyde, and those derived from clove, coriander, and star anise. Thymol-cinnamaldehyde blends (108) and oregano EO (109) improved body weight, and correlated with lower FCR (110). Notably, a commercial PFA blend of carvacrol, cinnamaldehyde, and capsicum oleoresin received EU authorization for broiler performance enhancement, with meta-analyses confirming consistent improvements in weight gain, FCR, and mortality reduction (111, 112). Future research should prioritize elucidating structure–activity relationships and optimizing delivery systems to maximize consistency in livestock production.

Following a ban on AGPs, a Brazilian broiler farm faced high mortality, wet litter, and a poor FCR (113). The introduction of a PFAs (blend of thymol, carvacrol, and cinnamaldehyde) at 150 g/ton of feed resulted in a 4-point FCR improvement (1.68 to 1.64), significantly drier litter, and reduced intestinal lesions, demonstrating enhanced gut health and nutrient digestibility (114). A 1,200-sow operation in Spain faced severe post-weaning diarrhea in piglets, resulting in high mortality, growth checks, and heavy reliance on antibiotics. Pre-weaning liquid supplement with carvacrol and anise oil from day five stimulated early gut development, followed by a post-weaning diet for 4 weeks containing a micro-encapsulated PFAs high in carvacrol and cinnamaldehyde resulted in 60% decrease in diarrhea, 12% increase in Average Daily Gain in the first 4 weeks post-weaning, and cut 70% use of antibiotics (115). A 500-cow US dairy farm feed blend of PFAs (capsicum, cinnamaldehyde, and eugenol) to the total mixed ration resulted in selectively inhibiting lactate-producing bacteria to stabilize rumen pH and enrich the population of fibrolytic bacteria. The enhanced ruminal environment directly boosted productivity, increased average milk yield by 1.5 kg/cow/day and 0.3% increase in milk fat (258). The economic return from these performance gains fully offset the additive’s cost, providing a viable non-antibiotic strategy to restore profitability. Although, PFAs are low risk to drive AMR, but some bacteria can develop non-genetical tolerance through general response pathways, efflux pump upregulation, or biofilm formation which may result in the selection of more broadly resistant strains.

### Hyperimmune egg yolk antibodies (HEYAs)

Hyperimmune egg yolk antibodies (IgY), produced by repeatedly immunizing layers with specific antigens and subsequently harvesting antibodies from their yolks, are commonly employed in preventing and treating various enteric diseases in animals (116). However, limited research on their efficacy as antibiotic growth promoter (AGP) alternatives for enhancing poultry growth and feed efficiency (117). In livestock, maternal antibodies transfer to offspring to improve productivity. Pimentel et al. (118) observed increased body weight in three-week-old chicks from hens injected with jack bean urease, suggesting that maternally derived urease antibodies may enhance growth by inhibiting bacterial urease and reducing intestinal ammonia production. With advancements in IgY technology, subsequent research explored dietary antibody supplementation to enhance host immunity or performance (Table 3) (119). Many studies utilized antibodies targeting immune-modulatory molecules, given that immune activation suppresses growth—likely due to inflammation-induced release of anorexigenic neuropeptides like cholecystokinin (CCK) and neuropeptide Y (NPY) (120).

**Table 3:** Hyperimmune egg antibodies as alternative antimicrobials.

Antibody	Species	Pathogen/Physiology	Findings	References
Multivalent egg yolk IgY	Chicken	*Eimeria tenella*	↑ weight and anti-coccidial index, ↓ mortality, coccidial counts, lesions in the cecum, and shedding of the oocytes	(206)
Egg yolk antibody (IgY) powder	Layer	*E. coli*	150 mg/mL: ↓ proliferation by 1.18 CFU/mL	(207)
IgY powder	Broiler	Growth, immunity, intestinal morphology of *E. coli* (O78: K80)	0.1–0.4%: Decreases ileal bacterial counts and blood infection parameters	(208)
Chicken egg-yolk derived antibody against 5 *C. jejuni* CAPs	Hen	*C. jejuni*	↑ α-*C. jejuni* CAP-specific IgY levels in immunized hens; ↓ hepatocellular carcinoma cells	(209)
IgY hyperimmune serum	Broiler	*C. jejuni*	↑ mucosal clearance; ↓ bacterial count and transmission	(210)
Polyclonal IgY	Hubbard broiler chicks	IBDV	↓ morbidity, mortality, and virus-specific lesions	(211)
Hyperimmune egg yolk	Layer Hens	*C. jejuni*	↓ *Campylobacter*-colonization, ↓ in *C. jejuni* counts	(212)
IBDV-specific IgY serum	Chicks challenged with IBDV	IBDV	Serum titer more than 4,000 was effective	(213)
Hyperimmunized yolk antibodies raised in hens	Piglets	PEDV and TGEV	100 and 88% cure rate against TGEV and PEDV	(214)
HRV received specific IgY antibodies	Gnotobiotic Piglets challenged with infectious Wa G1P	Human Rotavirus	Titer of 4,096 for 9 days: full protection against diarrhea, ↓ shedding of the virus	(215)
Bovine RV-specific IgY in egg yolk	Newborn calves	Bovine rotavirus	10^5.85^ FFU: 80% protection against diarrhea	(216)
Enterobactin-specific hyperimmune egg yolk IgY		Immunity	The immune response generated against Gram-negative infections	(217)
IgYs raised against Omp34	Mice	*Acinetobacter baumannii*	Passive immunization, ↓ bacterial load in various organs	(218)
IgY raised against soluble tachyzoite antigens	Hens and Mice	*Toxoplasma gondii*	↑ Recognition of parasite antigens	(219)
Supracox®	SPF Leghorn Chicks	*Eimeria tenella*	120 mg/bird: ↑ viability and weight gain; ↓ oocyst output per gram of feces, cecal tissue, and cecal lesions	(220)
Ovotransferrin PC2 from egg white of hen	Rhode Island Red Hens	Immunological activity	Antigen specificity and high antibody titers are similar to IgY	(221)
Chicken IgY specific to GST-GAM56	Chicken	*Eimeria maxima*	↑ weight; ↓ oocyst output and intestinal lesions	(222)
IgY extracted from hyperimmunized chickens	Rats	*T. evansi*	Pre-patent period, endurance and survival were enhanced	(223)

**Figure 5:**
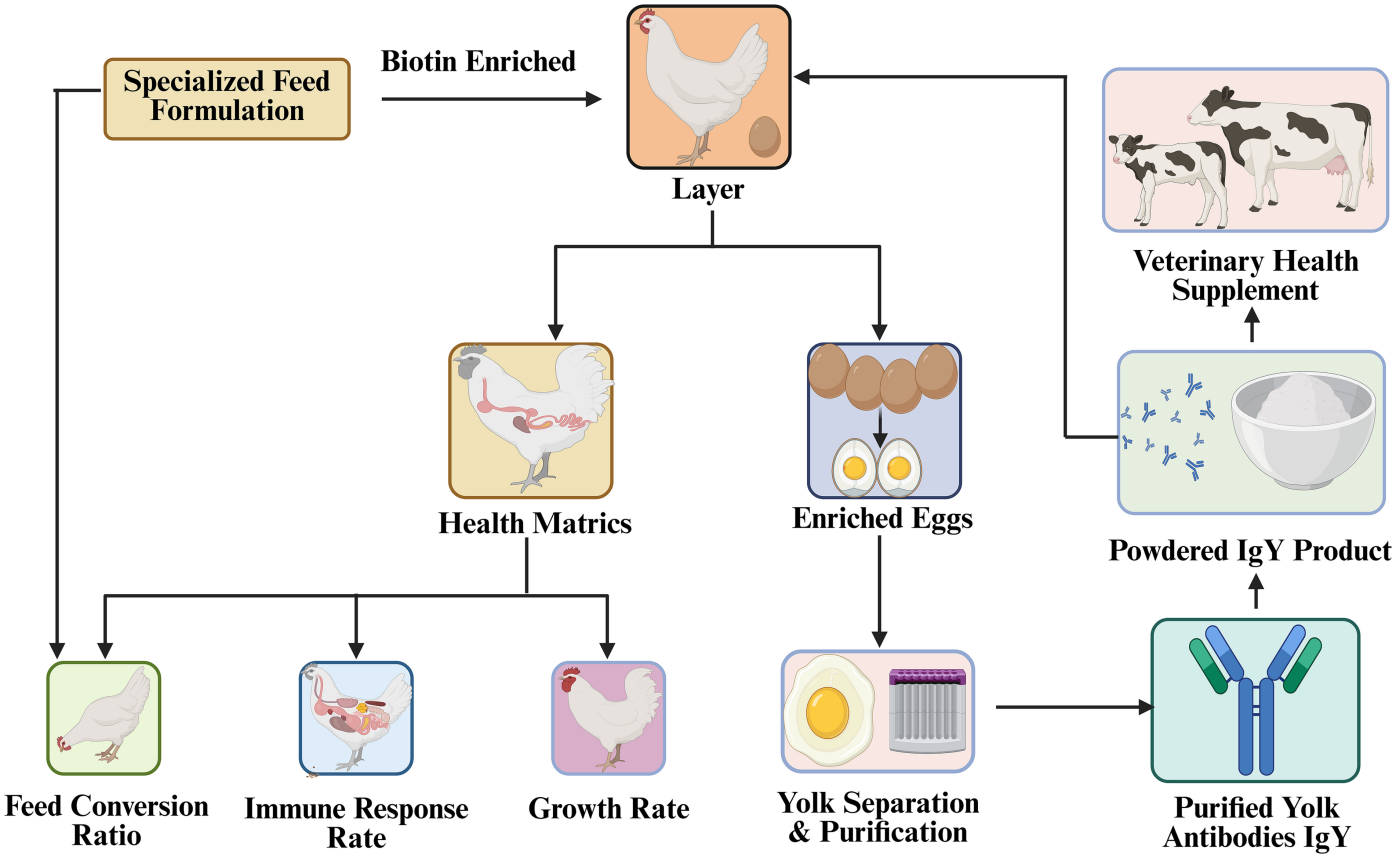
Value-added eggs as therapeutic antibodies. Eggs yolks of hens fed on biotin-enriched specialized diet are rich in IgY and used as veterinary health supplements or passive immunization therapies.

Cook (117) demonstrated that feeding broilers with non-approved HEYA powder at the rate of 2% against Cholecystokinin (CCK) or neuropeptide Y (NPY) for 3 weeks improved body weight and feed efficiency. Specifically, dietary inclusion of CCK antibody-enriched egg powder (0.25 g/kg) enhanced FCR by 13 points compared to controls (Figure 5). Similar improvements were noted in chicks fed at 2% NPY antibody powder namely NovaGrow®, a FDA approved safe feed for animals, showing a 9% increase in weight gain and an 8-point improvement in FCR by 3 weeks (117). Supplementing diets with 0.1% BIG™ (Bio-Immunoglobulin), approved and commercially available, targeting phospholipase A2—an enzyme involved in pro-inflammatory eicosanoid synthesis—for 3 weeks increased broiler weight gain by 5.4% and improved FCR by 6.2 points (117). IgY technology offers several advantages: high-yield antibody production in hens, non-invasive collection, low toxicity, environmental compatibility, and no risk of resistance development. Despite promising preliminary results, further research is necessary before implementing egg antibodies as a growth-enhancing strategy in poultry.

### Genomic medicines (GMs)

Growing global concern over antibiotic resistance in livestock production have spurred the exploration of alternative strategies. Among the most significant advancements is the application of genomic medicine—leveraging molecular-level insights to diagnose, understand, and manipulate biological systems in animals. This field encompasses gene editing (e.g., CRISPR/Cas9), genome-wide association studies (GWAS), transcriptomic profiling, and vaccinomics (Table 4), all aimed at enhancing disease resistance, improving vaccine efficacy, and reducing reliance on antibiotics (121). A key application of genomic medicine is the identification and selection of genetically disease-resistant animals. By pinpointing favorable alleles associated with resistance to pathogens such as *E. coli*, *Salmonella*, or *Clostridium perfringens* (122, 123), breeders can integrate these traits into selection programs, fostering herd immunity. This approach reduces disease prevalence, diminishes the need for prophylactic or therapeutic antibiotics, and supports One Health-oriented, sustainable animal production.

**Table 4:** Genomic medicine as an alternative to antibiotics in poultry and livestock production.

Aspect	Techniques	Examples	Species	Benefits	References
Gene editing	CRISPR-Cas9, TALENs	Editing *CD163* to resist PRRS	Pig	Immunity, disease resistance	(224)
Disease resistance breeding	GWAS, marker-assisted selection	Salmonella, Mycoplasma, *E. coli resistance*	Chicken	Natural selection	(225)
Vaccinomics	Genomic profiling, transcriptomics, epitope prediction	DNA/RNA vaccines against avian influenza, Avian retroviruses, necrotic enteritis	Avian	Customized vaccines	(226)
Diagnosis and prediction	Genomic diagnostics, SNP chips	Mastitis susceptibility	Cattle	Early detection and prediction	(227)
Microbiome engineering	Metagenomics, Host-microbiome interaction	Selection of favorable gut microbiota	Chicken	Improve gut health without antibiotics	(228)
Precision livestock medicine	Integrating genomics data for precise farming technologies	Individual health protocols based on genomic makeup	Livestock	Real-time health monitoring and targeted interventions	(229)

Furthermore, gene-editing technologies like CRISPR enable direct genome modifications to enhance immunity or disrupt pathogen replication. For example, editing the CD163 gene in experimental pigs has conferred resistance to porcine reproductive and respiratory syndrome (PRRS), a disease historically controlled with antibiotics (124, 125). Similar strategies are being investigated in poultry to combat necrotic enteritis and avian influenza, offering precise, permanent solutions to infectious diseases without environmental trade-offs. Additionally, vaccinomics—guided by immune response network theory—facilitates the development of tailored vaccines optimized for specific breeds or regional pathogens (126). These next-generation vaccines induce stronger, more targeted immune responses, reducing the incidence and severity of bacterial infections in livestock and poultry (127). When integrated with precision livestock farming, genomic medicine paves the way for a more resilient, antibiotic-independent production system.

### Antimicrobial peptides (AMPs)

**Figure 6:**
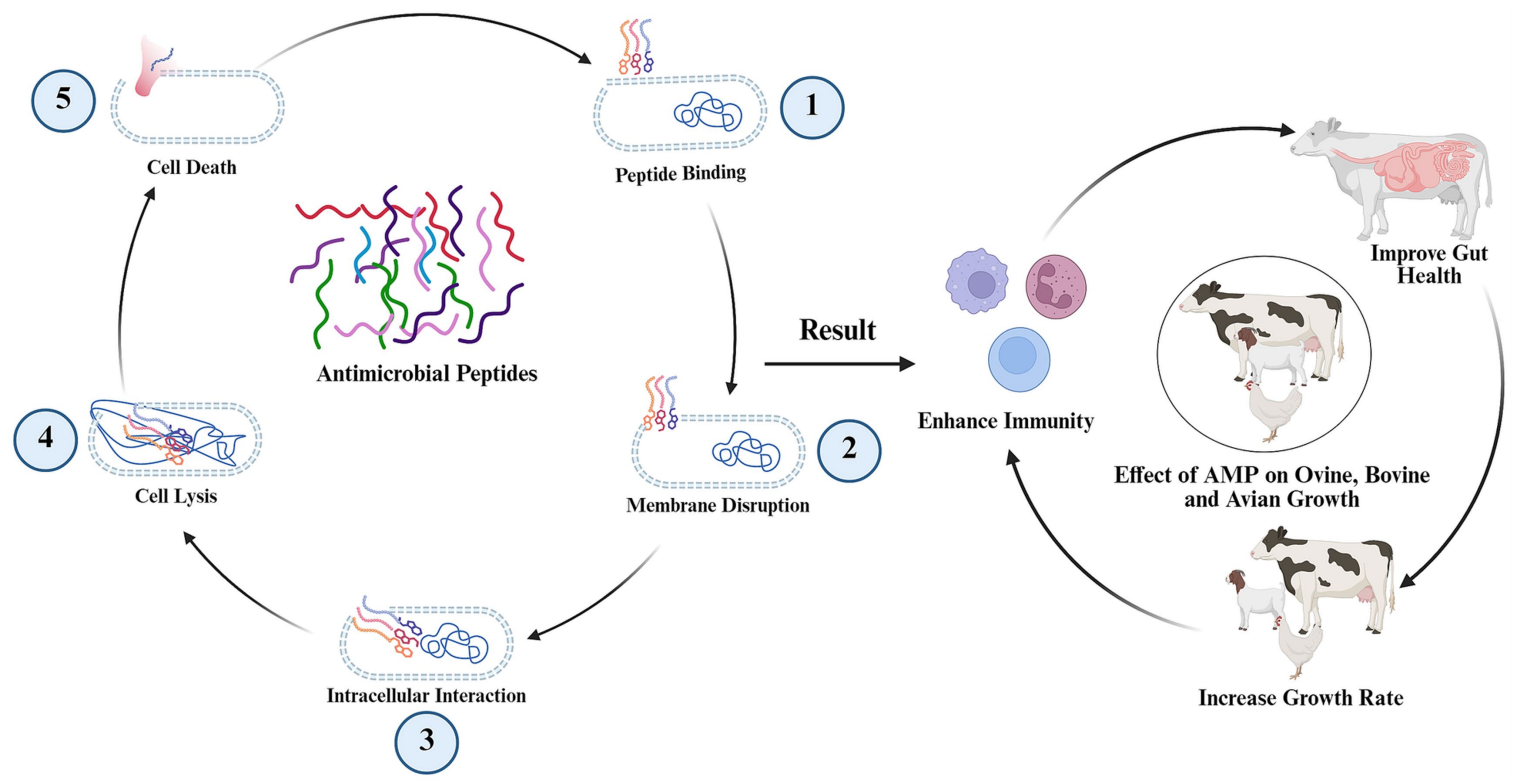
Antimicrobial peptides (AMPs) in livestock production. Antimicrobial peptides are immuno-modulators which degrade outer membrane and disrupt intracellular mechanisms of pathogens to avoid disease incidence. So, AMPs foster a healthy gut environment, improve growth performance and feed efficiency in household animals.

AMPs are small, gene-encoded germicidal molecules which exhibit broad-spectrum activity against diverse pathogens, including bacteria, fungi, parasites, and enveloped viruses (128). Mature AMPs typically comprise 12–100 amino acids, characterized by cationic and hydrophobic residues that confer an amphipathic structure, enabling interactions with negatively charged microbial membranes and other cellular targets (Figure 6). Over 2,600 endogenous AMPs have been identified, alongside numerous synthetic analogues (APS Database)^1^ (129). While research on AMPs in livestock has predominantly focused on their defensive role against infectious pathogens, emerging studies suggest their potential as alternatives to AGPs. For instance, dietary supplementation with the chimeric peptide cecropin A (1–11)-D(12–37)-Asn (CADN) enhanced weight gain, feed efficiency, jejunal and cecal villus height, and reduced aerobic bacterial counts in digesta (130). Similarly, the CAMA peptide (a cecropin A-magainin 2 hybrid) improved growth performance, nutrient retention, gut morphology, and microbiota composition (Figure 3) (131). Naturally derived AMPs—isolated from porcine intestines or rabbit sacculus rotundus—also enhanced growth, nutrient absorption, and mucosal immunity (e.g., secretory IgA and intraepithelial lymphocytes) in poultry (132).

Bacteriocins, a subclass of AMPs, are ribosomally synthesized antimicrobial peptides produced by bacteria and archaea, exhibit narrow-spectrum activity against phylogenetically related strains. Initially recognized as food preservatives, they are now considered pivotal in probiotic selection (133). To date, 177 bacteriocins from 31 bacterial and archaeal species have been documented (BACTIBASE).^2^ For example, Divercin AS7 (*Carnobacterium divergens*) is in research phase and not commercially approved for food or feed which has shown improved growth efficiency, nutrient digestibility, and gut microbiota balance while reducing digesta pH in broiler at the rate of 10^7^ CFU/g of feed (134). Similarly, Nisin (Nisaplin®, *Lactococcus lactis*) is FDA-approved bacteriocin for food not feed, it modulated gut microbiota by suppressing Bacteroides and Enterobacteriaceae without affecting *Lactobacillus* or *Clostridium perfringens* at the rate of 3,000 mg/kg diet (135). Albusin B (*Ruminococcus albus*) is another non-approved prebiotic which enhanced growth performance, lipid metabolism, antioxidant capacity, and *Lactobacillus* proliferation in broiler at the rate of 3% of the diet (136, 137). Although AMPs demonstrate promise in replacing AGPs, but their production cost is very high, may have potential resistance development by microbes altering surface charge, enacting proteolytic degradation, and upregulating efflux pumps. Consequently, the utilization of AMPs as growth promoters risks inadvertently selecting for strains with enhanced virulence and broadened intrinsic resistance, thereby compromising both innate immunity and last-line antibiotic therapies.

### Bacteriophages

Bacteriophages are highly specific viruses that exclusively infect and lyse bacterial cells through the production of endolysins, while remaining inert to eukaryotic cells in plants and animals. Their precise bactericidal activity has led to applications in both prophylactic and therapeutic interventions against bacterial infections in humans and livestock (138). Extensive research has demonstrated their efficacy in controlling foodborne pathogens within agricultural production systems (139). Emerging evidence suggests potential growth-promoting effects when incorporated into poultry diets. In laying hens, dietary supplementation with zootechnical approved feed additive namely Bafasal®, a *Salmonella*-targeting bacteriophages cocktail, at the rate of 0.035–0.05% (350–500 g/metric ton of feed) significantly enhanced egg production (140). Similarly, broilers receiving 0.10–0.15% of lytic bacteriophage cocktail targeting *Salmonella enterica* serovar Enteritidis feed supplementation, and 0.5 g/kg bacteriophage cocktail targeting *Clostridium perfringens* feed formulations exhibited significantly improved body weight gain and FCR (Table 5) (141, 142). SalmoFresh® by Intralytix^3^ and Preforpro® by Deerland Probiotics and Enzymes^4^ are FDA approved for food and feed additive for poultry, human and pets, respectively. While these findings indicate bacteriophages are potential antibiotic alternatives, but their application is very less due to limited host range because few host-phage receptor binding proteins exists. Bacteriophages impose strong selective pressure for the evolution of phage resistant bacteria via modification to cell surface receptors, i.e., lipopolysaccharides and porins. Further investigations are required to fully elucidate the growth-promoting mechanisms of bacteriophages and optimize their practical application in commercial poultry production systems. The regulatory landscape for phage therapy in animals is still evolving due to their variable nature, complex characterization, efficacy trials, intellectual property rights, and environmental impact assessment.

**Table 5:** Efficacy of various bacteriophages as alternative antimicrobials.

Bacteriophage	Species	Age (Days)	Pathogen	Findings	References
SalmoFree® (CJø01)	Layer Hen	42	*Salmonella* Gallinarum	10^6^ PFU/kg: <5% mortality vs. 30% (control)	(230)
ΦCJ07	Chicken	1	*Salmonella enteritidis*	5 × 10^7^ CFU/g: ↓ colonization	(231)
Siphovirus PSE	Quail	7	*Salmonella enteritidis*	10^8^–10^9^ PFU/mL: ↓ coli-bacilli and aerobe counts in vivo	(232)
ΦF61E, ΦF78E, ΦF258E	Chicken	730	*E. coli*	5 × 10^7^ and 10^9^ PFU/mL: ΦF78E ↓ 43% morbidity, ↓ 25% mortality	(233)
Coliphage cocktail	Chicken	21	*E. coli*	1,000 MOI: lysis of several phages	(234)
ΦCcoIBB37, ΦCcoIBB12, ΦCcoIBB35	Chicken	31	*C. colli* and *C. jejuni*	5.8 × 10^6^ CFU/g: ↓ fecal bacterial titer → 30-fold ↓ campylobacteriosis incidence	(235)
*Staphylococcus* phages (B4 and M8)	Bovine mastitis	730	*S. aureus*	10^8^–10^9^ PFU/mL M8: effective vs. MDR/MRSA/biofilm strains	(236)
KpV74 and Dep_kpv74 depolymerase	Mice	42–56	*Klebsiella pneumonia*	10^8^–10^9^ PFU/mL: ↓ behavioral effects and toxicity	(237)
Cocktail of 6 bacteriophage strains	Dog	Species-dependent	*P. aeruginosa*	1 × 10^5^ PFU/ml: ↓ counts 48 hpi and no toxicity	(238)
4 new canine phages	Dog	7–42	*S. pseudintermedius*	10^8^–10^9^ PFU/mL: lytic for all MRSP and 16–28% of MSSP isolates	(239)
Phage from *S. typhimurium* infected pigs	Pig	21–60	*Salmonella typhimurium*	10^7^–10^9^ PFU/mL: ↓ 93.3 to 56.6%	(240)
CJ12	Pig	7–42	*E. coli*	10^6^ and 10^8^ PFU/g: ↓ diarrhea	(241)
P22 phage	Chicken	7–42	*Salmonella*	10^8^–10^9^ PFU/mL: ↓ motility in the gut	(242)
Pbunavirus PB1-like phage	Mice	42–56	*P. aeruginosa*	10^8^–10^9^ PFU/mL: 100% survival	(243)
ϕNH-4 and ϕMR299-2	Mice	42–56	*P. aeruginosa*	10^8^–10^9^ PFU/mL: killed in the murine lung and pulmonary cell line biofilm	(244)
Phage cocktail	Pigs	21–28	*Salmonella typhimurium* (*γ*423216)	5 × 10^9^ PFU: ↓99.9% in tonsils, ileum, and caecum	(245)
Bacteriophage cocktail	Pigs	180–730	*Salmonella*	≥10^9^ PFU/ml: ↓ fecal bacterial count and *Enterobacteriaceae* species, normal microflora undisturbed	(246)
GRNsp6, GRNsp8, GRNsp51 cocktail	Chicken	7–42	*Salmonella enteritidis*	10^9^ PFU: ↓ intestinal colonization and lowered duodenal mRNA expression of IFN-γ, IL-6, and IL-1β	(247)
Coliphages from goat kids	Goat	1–14	EPEC MDR strains	10^8^–10^9^ PFU/mL: Potent antibacterial efficacy in vitro	(248)
Romulus, Remus, ISP	Mice	180–730	Mastitis	10^8^–10^9^ PFU/mL: ISP phage in vivo resulted in partial improvement of mouse mastitis at 48 HPI	(249)
Pbunavirus PB1-like	Dog	42–56	*P. aeruginosa*	2 × 10^8^ pfu/mL: Clear inhibition of the occurrence of the phage-resistant variant	(250)
ΦR18 cocktail	Mouse	7–42	*P. aeruginosa*	10^8^–10^9^ PFU/mL: ↓ bacterial and keratitis prevention	(251)
EB1.ST11, EB1.ST27, STA1.ST29 (1:1:1)	Bovine	7–42	*S. aureus*	10^8^–10^9^ PFU/mL: 66% of isolates were lysed	(252)
PlySs2 and PlySs9 endolysins from *S. suis* serotype-2 and −9	Bovine	30–180	*S. uberis*	10–100 μg/mL: ↓ OD, and 100% lysis	(253)
Sewage water origin SA phage	Bovine	180–730	*S. aureus*	10^8^–10^9^ PFU/mL: ↓ growth	(254)
Active recombinant endolysin from IME-SA1	Bovine	180–730	*S. aureus*	10–100 μg/mL: effective against mild clinical mastitis	(255)
SAJK-IND, MSP	Bovine	180–730	*S. aureus*	0.01 MOI: 100% susceptible to AJK-IND and 40% to MSP	(256)
HY-133	Livestock	180-730	Genetically diverse MRSA livestock isolates	5 × 10^5^ CFU/mL: livestock associated-MRSA having mecA, mecB, and mecC were susceptible	(257)

### Clay minerals (CMs)

Phyllosilicate clays are layered aluminosilicate minerals composed of tetrahedral and octahedral sheets interconnected through hydrogen bonds or cationic bridges (143). Naturally occurring clays—including bentonite, zeolite, and kaolin—represent complex mixtures of these minerals with heterogeneous chemical compositions (143). Their unique structural properties confer exceptional adsorption capacity, enabling the binding of aflatoxins, enterotoxins, heavy metals, viral particles, and plant metabolites. The extent of adsorption is governed by clay chemistry, particle morphology, surface characteristics, pH, dosage, and exposure duration (87). While extensive research has documented the antimicrobial and detoxification properties of clays in livestock (144), their growth-promoting effects remain less characterized.

Copper-bearing montmorillonite supplementation significantly improved broiler growth performance, reduced *E. coli* and *Clostridium* spp. colonization, and enhanced intestinal digestive enzyme activity (145). Similarly, dietary hydrated aluminosilicate (5 g/kg) increased body weight gain and elevated lactate dehydrogenase and amylase activity in broilers (146). Comparable benefits were observed with kaolin, bentonite, and zeolite supplementation (147). Clinoptilolite supplementation enhanced systemic antioxidant status, as evidenced by increased hepatic glutathione peroxidase, catalase, and superoxide dismutase activity, alongside reduced malondialdehyde levels (148). However, growth performance responses have shown variability across studies, with some reporting neutral effects (148). The growth-promoting mechanisms of clay minerals could be due to toxin sequestration, gut function modulation and nutrient utilization (147). Further research is warranted to evaluate clays as viable AGP alternatives and investigate potential synergistic interactions with other feed additives.

### Trace minerals (TMs)

Essential trace minerals such as Cu, Zn, Fe, Se, and Mn serve as co-factors for numerous enzymatic and biosynthetic processes which play critical roles in maintaining livestock health and metabolic function directly influencing growth performance and physiological development (149). Traditionally supplemented as inorganic salts (carbonates, chlorides, oxides, sulfates) or organic complexes, their application at supernutrition levels has gained prominence for performance enhancement in modern poultry production. Cu is a vital catalytic element in connective tissue formation, hemoglobin synthesis, and angiogenesis (150), demonstrates significant growth-promoting effects when supplemented at 125–250 mg/kg diet in forms such as sulfate, citrate, or carbonate (151). Comparative bioavailability studies indicate that organic complexes like cupric citrate (resulting in 9.1% weight gain increase) can outperform inorganic sources like cupric sulfate (4.9% increase) in broilers (152), with tribasic copper chloride similarly enhancing daily growth and carcass yield (153).

Zn, an essential micronutrient for cellular proliferation, immune modulation, and oxidative protection (154), has shown variable effects. For instance, ZnSO_4_ supplementation at 80 mg/kg improved body weight without affecting feed efficiency (155), while other studies report its primary benefits as immunostimulatory rather than directly growth-promoting (156). The antimicrobial properties of both Cu and Zn contribute to their efficacy, modulating gut microbiota by reducing pathogenic and commensal bacterial populations (157). However, mineral supplementation presents environmental pollution such as excessive metal accumulation in soil and water systems (155), coupled with the emergence of metal-resistant enteric bacteria exhibiting cross-resistance to antimicrobials (157). The EU implemented a regulatory ban on pharmacologic Zinc Oxide (ZnO) in livestock feed in 2022 (158), driven by its environmental persistence and role in co-selecting AMR (159), mandates an industry paradigm shift in trace mineral strategy from blunt, high-dose disease control toward precision nutrition that leverages enhanced bioavailability to build host resilience and mitigate these externalities (160).

## Conclusion

The growing consumer demand for antibiotic-free animal products has necessitated the development of effective alternative growth promoters (AGPs) capable of maintaining optimal health and production efficiency in food animals. AGPs such as probiotics, prebiotics, synbiotics, dietary organic acids, dietary enzymes, phytogenics, hyperimmune egg yolk antibodies, genomic medicines, antimicrobial peptides, bacteriophages, and clay and trace minerals have demonstrated potential in livestock production. Notably, AGPs such as blended organic acids (BOAs) and trace minerals are highly cost-efficient, probiotics and bacteriophages are moderate to high, bacteriocins are low, hyperimmune egg yolks are low to moderate, and essential oils are variable. Scalability of BOAs, probiotics and minerals is excellent, essential oils and bacteriophages is good, while hyperimmune egg yolk and bacteriocins is poor. One health implications of all AGPs is highly positive except trace minerals which cause environmental pollution. Efficacy of AGPs remain inconsistent, with performance outcomes varying significantly across production systems. This variability underscores the importance of selecting alternatives tailored to specific operational requirements. This further demands mechanistic elucidation – defining precise modes of action to predict and standardize effects, delivery optimization – advancing targeted-release technologies (e.g., microencapsulation) to enhance bioavailability and site-specific activity, and synergistic formulation – evaluating combinatorial approaches to achieve antibiotic-like efficacy through additive or synergistic interactions for antibiotic alternatives. Ultimately, sustainable reductions in antibiotic use will depend on integrating optimized alternative blends with improved management practices, ensuring both animal productivity and welfare in antibiotic-free production systems.

## Footnotes

1
https://aps.unmc.edu

2
http://bactibase.pfba-lab.org

3
www.intralytix.com/product/3?e=SalmoFresh

4
www.bacteriophage.news/database/deerland-probiotics-enzymes
